# Hospital and Institutional News

**Published:** 1920-01-31

**Authors:** 


### HOSPITAL AND INSTITUTIONAL NEWS.

### A ROYAL MESSAGE

In response to a message of homage from the patients of the Great Northern Central Hospital, who were entertained at a concert on Tuesday, January 13, the expenses of which were defrayed by His Majesty, the following reply was sent: "The King has received with much pleasure your telegram and hopes that the patients of the Great Northern Central Hospital have spent a pleasant evening together." This hospital has also received a contribution of fifty pounds from the Royal Naval Canteen Committee, Dover, being donations subscribed by petty officers and ratings attached to the Dover Patrol.

### DEATH OF DR. LYSTER.

We deeply regret to record the death of Dr. Cecil R. C. Lyster, of 70 Wimpole Street, W., which adds one more name to the list of those who have fallen victims to the effects of *x*-ray research. Dr. Lyster, who was sixty years of age, was for seventeen years in charge of the Middlesex Hospital Electro-therapeutic Department, and his pioneer work in relation to *x*-rays, more particularly to the treatment of cancer, is well known to all. It was through exposure to the rays in the early days of this science that his malady was contracted, but in spite of great suffering he persisted in following up his valuable work to the end.

### DEATH OF LONDON FEVER HOSPITAL SECRETARY.

Major W. Christie, for so long Secretary of the London Fever Hospital, Liverpool Road, N., on his retirement last year, was succeeded by Mr. W. J. H. Brand, who died on Saturday last. We understand that Mr. Brand, a Brevet Major in the London Scottish, was not previously known in the hospital world, but from the promising commencement he had made at the London Fever Hospital, a useful hospital career has been cut short. Mr. Brand was in his fiftieth year of age, and died of heart failure following upon scarlet fever after one week's illness. In connection with his funeral it was asked that, instead of sending flowers, friends might make small offerings to the special appeal fund of the hospital, which had been launched just before the Secretary was taken ill.

### PRODUCTIONS OF DISABLED MEN.

Those who have a taste for beautiful or quaint things should visit that amazing business establishment of "The Brownies," at 12 Orchard Street, Portland Square. Everything the shop contains is the work of soldiers and sailors disabled in the war, their widows and dependants. It is perhaps but slight consolation for the suffering of so many men that latent artistic gifts have been aroused during the long days of convalescence. Nevertheless, the imagination and ability displayed by the work of their hands are no less astonishing than gratifying. There are in this shop pictures, lacquer work, beads, carving of excellent quality, and a profusion of toys which range from the extreme bizarre to wonderfully fine mechanical models. This wide variety of articles has been brought together in London from hospitals and curative establishments all over the country, and Mrs. L. Bulteel, founder of the "Brownies," was responsible for providing the market for them in Orchard Street. Those who buy get good value for their money, and the satisfaction of helping those who have been handicapped by their war sacrifices, is thus given free of charge. Perhaps the most remarkable feature about the work of these disabled men is that it is nearly all designed to give pleasure or amusement—as though, in nearly giving their life, they had discovered what a good thing life really is.

### THE REAL POINT IN THE CHARGE AGAINST ST. THOMAS'S.

When the full facts have been disclosed the charge, which has been freely circulated, that the authorities at St. Thomas's Hospital had been parties of callousness falls to the ground. It was alleged at a meeting of the Lambeth Board of Guardians that a woman nearing her confinement after a partial operation by a doctor at her private residence had for six hours been driven in a taxi-cab, first to the Lying-in Hospital, York Road, and then to St. Thomas's Hospital. At neither place was there a vacant bed, and ultimately, after six hours in the taxi-cab, she reached Lambeth Infirmary, where the operation was successfully performed. In the first place the woman never was six hours in a taxi-cab. The only journey the woman took was from her home to Lambeth Infirmary, a journey which did not take half-an-hour. Sir Arthur Stanley, the treasurer of St. Thomas's Hospital, has given the facts so far as the hospital authorities are concerned. The patient's husband called at the hospital with a note from the doctor attending the case to ask whether the woman could be admitted; the answer regretted that the wards were full. Then the husband went to the Lying-in Hospital, where he was informed there was no vacant bed, and eventually he found his way to Lambeth Infirmary. Later his wife was taken there in the taxi-cab.

### PECULIAR PROBLEM OF MATERNITY BEDS.

Sir Arthur Stanley naturally emphasises the great need for special beds to deal with maternity cases in the hospitals. Most of them are bespoken weeks before they are occupied, and to take a bed which may be occupied by another patient in a few days would naturally cause great inconvenience and perhaps suffering. The same argument applies to the Lying-in Hospital. Some time ago the Lambeth Board of Guardians were considering whether a special ward should be reserved for expectant mothers who could afford to pay for their confinement, but who could not be properly attended at home. Nothing has, however, come of the suggestion. Mr. Frank Briant, M.P., the chairman of the Lambeth Board of Guardians, has now admitted that the first history of this case was incorrect, but is of opinion that hospital accommodation in London is on a wrong basis when it is possible for so serious a case "to be bandied about from one institution to another." In his anxiety that the Ministry of Health shall co-ordinate all systems for institutional treatment, Mr. Briant will remember that the first thing will be to provide the lacking accommodation, and could not the Lambeth Board of Guardians do this itself if the members were serious in the matter?

### THE KENT PANEL DOCTORS.

In the County of Kent dissatisfaction of the public with the panel doctors, and of the panel doctors with the Insurance Commissioners, is not, so far as we know, more acute than in other places; but that of the doctors is certainly more vocal and better organised than elsewhere. The Kent Local Medical and Panel Committees have sent us a long criticism of the latest phases of National Insurance, together with an explanation of their attitude in case a breach occurs which causes them to withdraw from the panel; they lay emphasis on the fact that they have no intention of refusing to treat the sick. The criticism of their manifesto which onlookers are most likely to make is that they try to prove too much. It is, no doubt, true enough that many of the regulations do not make for effective treatment of the insured, and may even hinder it; and also that some of the permanent officials care little or nothing about the patients, but only about making themselves safe and snug in their posts by a liberal expenditure of red tape. But it is not true to represent the panel doctors, one and all, as conscientious and highly efficient men in love with hard work; at least, it is not so in London, and we doubt whether it is in Kent. Such men there are, of course, on the panels in plenty; but they are outnumbered (in the Metropolis) by the ignorant, the careless, and the downright incompetent. The greatest drawback to a State medical service is the certainty that this type will be encouraged (as it already is under the existing panel system) and the better types correspondingly discouraged.

### FINANCES OF THE METROPOLITAN ASYLUMS BOARD.

The expenditure statement of the Metropolitan Asylums Board for the year ending March 31, 1919, is a document of fourteen foolscap pages of figures relating to the thirty or more hospitals and institutions controlled by the Board. The gross expenditure was £1,469,512, as compared with £1,331,754 in the previous corresponding twelve months. Of this huge sum the hospitals accounted for £431,988 and the asylums for £372,544, as against £404,211 and £301,970 respectively for the year ending March 31, 1918. From the mass of detail before us it is possible to mention only some of the more striking points The maintenance of inmates amounted to £251,265, an increase of over £20,000; salaries and wages, £414,990, comparing with £323,745; maintenance, etc. of staff, £235,723, higher by £27,000 than the previous year. The only item which is but slightly higher is rates and taxes. It will be noticed that the experience is, as elsewhere in the institutional world, that by far the larger increase in expenditure is in respect of the remuneration of workers.

### SALES OF SURPLUS MATERIAL.

The Prince Alfred Hospital, Sydney, has recently been able to carry out a successful financial operation by purchasing at auction a large quantity of material, disposed of by the military authorities, and consisting of drugs, enamelware, and rubber and other goods such as are in daily use at a hospital. Altogether £250 was spent, and an examination shows that the market prices are about three times as great as the sum paid. We have heard some whispers as to the sale of similar articles in London under the hammer, which indicate that our home hospitals are not so fortunate as the Prince Alfred. Very substantial prices have been tendered for articles offered, but their purchase has not come about. We have heard of an instance where, as a test, full retail value, less the cost of washing, was offered for a parcel of blankets which, however, did not result in a purchase. Either the reserve fixed by the Government was too high, or a syndicate was bidding whose business it was to frighten away the ordinary buyer. It does not seem unreasonable to make the suggestion that the Surplus Disposal Board should allow hospitals an opportunity of buying hospital articles at a fair price before offering the goods by auction.

### BANK STOCK AS AN INVESTMENT.

The net profits of the London County Westminster and Parr's Bank, Limited, for the past year, after providing for bad and doubtful debts, and all expenses, amount to £2,455,007. This sum, added to £377,560, brought forward from 1918, leaves available the sum of £2,832,567. The dividend of 10% paid in August last absorbs £494,969. A further dividend of 10% is now declared in respect of the £20 shares, and the maximum dividend at the rate of 12½% per annum on the new £1 shares will be paid. £1,000,000 has been set aside for investment depreciation, £100,000 transferred to the Bank's War Memorial Fund, in accordance with the resolution at the general meeting on January 30, 1919; £100,000 transferred to premises account; and £165,721 to reserve. brings the reserve up to £8,750,000, leaving a balance of £414,226 to be carried forward. The hospital is fortunate which possesses shares in banking.

### HOUSING IN INNER LONDON.

Public authorities in Inner London have a severe task in any attempt to solve the housing problem. Slums abound in every borough, and because all except urgent repairs have been postponed for the five years of the war, the property has gradually become worse, if that were possible. In Bermondsey, for instance, there are wide areas of slums, but unfortunately if a serious attempt was made to clear them thousands of people would be homeless. Dr. King Brown, the Medical Officer of Health, has done all that is possible without causing undue hardship or suffering, and further improvement now rests with the borough council. It is proposed to divide the borough into four areas. To ascertain the true state of affairs four assistant medical officers and eight additional sanitary inspectors shall be appointed. No new houses are to be built, and on any cleared area not more than twelve cottages to the acre are to be erected. Probably if a comprehensive scheme was provided to cover a number of years there might be a chance of making the borough a little more habitable, but to complete the work will require a sum of money that Bermondsey cannot provide.

### CARDIFF'S SUCCESS.

As reported in our columns last week, the Lord Mayor of Cardiff (Councillor G. F. Forsdike, J.P.) appealed for £150,000 for King Edward VII. Hospital, Cardiff. The success of his Lordship's appeal, reported last week, has been well maintained, no less a sum than £81,389 18s. 5d. having been received or promised up to Monday, 26th inst. Notable among these is the magnificent gift of some £6,000 from the ex-Service men of Cardiff and district. This forms the proportion of the Canteen Board's profits allocated to them for purposes of social welfare, and the unselfish action of the ex-Service men in handing the total sum over to King Edward VII. Hospital should stand out as a magnificent example to the whole country. The Lord Mayor of Cardiff has received many fine gifts, but it will be universally conceded that he has received no nobler one than this.

### GORDON MEMORIAL COLLEGE.

THE Gordon Memorial College at Khartoum has issued its seventeenth annual report with accounts, and it will probably be a matter of surprise to many of our readers to know that the educational work of the College and its valuable scientific and archæological investigations have proceeded in all tranquillity during the period of the war. The Egyptian Government largely depends on the College for candidates for the Government Service who have passed beyond the primary standard. Summer extension meetings for elementary vernacular school teachers have been recently instituted, and are becoming popular. There is a dramatic society among the students, a cinema, and a Boy Scouts' Brigade. Dr. Chalmers and his associates have devoted much time to the study of skin disease, and valuable entomological researches have been in progress.

### THE UNSHEPHERDED SUBSCRIBER.

A SUM of £35,000 for painting seems large, but the Edinburgh Royal Infirmary finds that this will be the cost of this one item at the present price of £600 for an ordinary ward. The financial outlook is so serious that it will have to find its remedy. The annual cost of maintaining the institution is about £77,000 a year, towards which about £42,000 is received. The annual deficit, which legacies can reduce but never cover, is therefore very serious. A league of subscribers has been started; one pound is the minimum subscription. The league has 1,700 members in Edinburgh, with a population of over 300,000. This means a membership of just over one-half per cent. The leagues which are successful are those whose members have a corporate sense, because they have a corporate activity. Is there no way in which subscribers to hospitals can be similarly provided, so that membership may come to mean more than the payment of a subscription with or without the appearance of a name in a list at the end of the report?

### SUBSCRIBERS AND LABOUR.

No greater disservice could be done to voluntary hospitals than to excite class feeling, but we sometimes wonder whether Labour is not doing so by the autocratic interference in which its members occasionally indulge. This feeling, which is generally below the surface, sometimes comes to light. We have lately read in the Provinces of private subscribers who have withdrawn their support on the ground that if Labour is to manage the local hospital exclusively it had better support it entirely. The argument, however regrettable, is logical. The Labour men who in industrial districts often provide most of the income for the local hospital are apt to regard the institutions as "theirs," and to forget that it was built by others, is partly supported by others, and derives its legacies exclusively from others. A hospital would be in a bad way which had only one class to depend on for support, and we wish that Labour would remember that no other class or person except itself regards its gifts to a hospital as conferring any right of entry or ownership. The penalty of having all things your own way is to have all the responsibility for them, and the need of the moment is for united effort from all who believe in the voluntary system without class-prejudice, which is as objectionable from Labour as from any other body. The working men in many places have already commenced to help to the utmost the voluntary hospitals in their districts up and down the country. The yield in England from this source is increasing in growing quantities year by year. This type of workman neither suffers nor permits autocratic interference by its members.

### QUEEN MARY'S HOSPITAL FOR THE EAST END.

MUCH quiet progress has been made of recent years at Queen Mary's Hospital for the East End, which the West Ham and Eastern General Hospital became not long ago. Needs and assistance keep within measure of each other, and the local war memorial has provided £30,000 for extensions. A further sum of £10,000 has been given to provide a maternity wing, which another £10,000 is needed to complete. The most pressing requirement is a nurses' home, which is expected to cost £30,000; the out-patient department needs extension. In other words, a sum of £50,000 is necessary, and a larger annual income must be found to maintain the extensions when they have been built. At the recent festival dinner, at which Mr. C. E. Leo Lyle, M.P., the hospital's Chairman, made an interesting speech, £5,000 was raised. The next step would appear to be a campaign for increased subscriptions. With an organisation under which a small army of voluntary workers might be organised to find and interest the fifty per cent. of the population who have not as yet given either service or money to the Queen Mary's Hospital. This plan has been approved by an increasing number of those responsible for the larger hospitals throughout England. The Red Cross, we have reason to hope, is likely to send a number of recruits of this description to Mr. Leo Lyle's assistance at Queen Mary's, should he desire to receive and organise their help in a practical way.

### AN INTERESTING BALANCE-SHEET.

In the year 1918 the Coventry and Warwickshire Hospital was in the unusually satisfactory position of balancing both sides of its current account, a remarkable feat, since the total on each side was over £28,000. In 1919, however, the income fell by about £4,000, and the expenditure increased by £2,000. Prices and the cost of redecoration explain the increase, and the cause of the fall in income is attributed to diminished receipts from the war. If this artificial loss, if one may so call it, can be made good, the general situation is encouraging, for last year not only did the Saturday Fund again contribute more—namely £8,350, a sum which may again increase if all the works fall into line by doubling their weekly contributions, as many have done—but general subscriptions were £400 higher. Donations were also larger. The immediate aim is to bring the contribution of the Saturday Fund to an average of £10,000. These sides of the income being in a healthy state, we would ask Mr. J. Warmell and the Secretary, Miss Hooper, if the employers are doing their share. For where no organisation of employers and heads of firms exists to agree to provide contributions on a *pro rata* scale in proportion to the sums raised by the workmen the old bad system of a casual cheque for a few guineas too often persists, which is a petty evasion of duty to a hospital where the men, when ill, are restored to health and activity.

### THE PROTEST OF THE WELSH MEMORIAL TO MR. AUSTEN CHAMBERLAIN

Six months ago the Welsh National Memorial Association applied to the Chancellor of the Exchequer for a grant or grants in aid of its work against consumption. At a recent meeting of the finance committee Mr. Austen Chamberlain's reply was read, and made such a bad impression that the Association has appointed a strong deputation to protest against its terms. A resolution was also passed to protest against the fact that the Chancellor had invited no representative of the Association to lay its case before him, to affirm its disappointment with his proposals, and to resolve to refer the matter not only to him, but to the Minister of Health. Telegrams were sent to both Ministers, asking them to receive a deputation from Welsh members of Parliament and from the Association's spokesmen. Every contributing authority in Wales, we understand, will be represented in the deputation.

### THE HOSPITAL TREASURY THEN AND NOW.

We cannot pass without appreciation the long record of service which has lately been completed by Mr. C. C. Sibthorp for Lincoln County Hospital. His retirement occurs at the end of forty years' service as honorary treasurer, a period which has seen a remarkable development of hospital finance in both of its departments. The great spurt which the voluntary hospital system received in the latter half of the nineteenth century was largely due to the interest which the hospital work aroused in the Royal Family, in particular in King Edward, during his long life as Prince of Wales. In 1880, to look back forty years, hospital expansion had begun. The harvest of work sown by Lister and Florence Nightingale was being reaped: in consequence, hospital expenditure was assuming its modern proportions; immense sums were being spent on bricks and mortar, the nurse was coming into the professional repute of a skilled and highly trained woman. The problem of raising money was therefore insistent. We wish that Mr. Sibthorp or some other treasurer of experience would sketch the change. For in the hospital world the details of forty years ago are already material for history. Colonel J. S. Ruston is the new hon. treasurer.

### LINCOLNSHIRE HOSPITAL EXTENSION.

A ten thousand pound scheme for the extension of the Lincoln County Hospital has just been adopted. It is part of a much larger scheme, which will be suspended for the present on account of the cost it must entail. Several departments are now to be added or brought up to date. A new out-patient and casualty department will account for £2,000, and the completion of the nurses' home for another £1,000. There are to be a new *x*-ray room, a pathological laboratory, and several necessary improvements in the domestic arrangements. Two electric lifts will absorb £2,000. Nearly £4,000 is in hand for the project, and it is hoped that by an arrangement with the Red Cross their gift of £10,000 conditional on the hospital providing a similar amount may be spent at once on an undertaking being given as to proceeding with the full scheme in the future.

### THIS WEEK'S DRUG MARKET.

On the whole prices are well maintained and the upward tendency of prices is noticeable in a number of instances. Salicylates have moved upwards again. Aspirin and amidopyrin are dearer. Quotations for borax and boric acid have been advanced. Makers of caffeine are busy and prices have an upward tendency. There is a large demand for citric and tartaric acid for export, and both are dearer. The demand is mainly for the United States, and the purpose for which these substances is required is the manufacture of temperance drinks; essence of lemon is in demand for the same purpose, and the price is much higher. Potassium permanganate seems to be getting scarcer rather than more plentiful, and is dearer. Belladonna leaves are offered at rather lower rates.

